# FDG-PET/CT and diffusion-weighted imaging for resected lung cancer: correlation of maximum standardized uptake value and apparent diffusion coefficient value with prognostic factors

**DOI:** 10.1007/s12032-018-1128-1

**Published:** 2018-04-09

**Authors:** Katsuo Usuda, Aika Funasaki, Atsushi Sekimura, Nozomu Motono, Munetaka Matoba, Mariko Doai, Sohsuke Yamada, Yoshimichi Ueda, Hidetaka Uramoto

**Affiliations:** 1 Department of Thoracic Surgery, Kanazawa Medical University, 1-1 Daigaku, Uchinada, Ishikawa 920-0293 Japan; 2 Department of Radiology, Kanazawa Medical University, Uchinada, Japan; 3 Department of Pathology and Laboratory Medicine, Kanazawa Medical University, Uchinada, Japan; 4 Department of Pathophysiological and Experimental Pathology, Kanazawa Medical University, Uchinada, Japan

**Keywords:** Lung cancer, Diagnosis, Diffusion-weighted magnetic resonance imaging (DWI), Magnetic resonance imaging (MRI), Positron emission tomography (PET), Prognostic factor

## Abstract

Diffusion-weighted magnetic resonance imaging (DWI) is useful for detecting malignant tumors and the assessment of lymph nodes, as FDG-PET/CT is. But it is not clear how DWI influences the prognosis of lung cancer patients. The focus of this study is to evaluate the correlations between maximum standardized uptake value (SUVmax) of FDG-PET/CT and apparent diffusion coefficient (ADC) value of DWI with known prognostic factors in resected lung cancer. A total of 227 patients with resected lung cancers were enrolled in this study. FEG-PET/CT and DWI were performed in each patient before surgery. There were 168 patients with adenocarcinoma, 44 patients with squamous cell carcinoma, and 15 patients with other cell types. SUVmax was a factor that was correlated to T factor, N factor, or cell differentiation. ADC of lung cancer was a factor that was not correlated to T factor, or N factor. There was a significantly weak inverse relationship between SUVmax and ADC (Correlation coefficient *r* = − 0.227). In analysis of survival, there were significant differences between the categories of sex, age, pT factor, pN factor, cell differentiation, cell type, and SUVmax. Univariate analysis revealed that SUVmax, pN factor, age, cell differentiation, cell type, sex, and pT factor were significant factors. Multivariate analysis revealed that SUVmax and pN factor were independent significant prognostic factors. SUVmax was a significant prognostic factor that is correlated to T factor, N factor, or cell differentiation, but ADC was not. SUVmax may be more useful for predicting the prognosis of lung cancer than ADC values.

## Introduction

Lung cancer is a heterogeneous cancer that has various patterns of progression and treatment responses. Positron emission tomography with 18-fluoro-2-deoxy-glucose (FDG-PET) has been widely adopted as the imaging method of choice in tumor staging. The maximum standardized uptake value (SUVmax) is a parameter of glucose uptake and usually indicates how aggressive the cancer is. FDG-PET/CT has helped differentiate malignant from benign pulmonary nodules [1]. However, FDG-PET/CT can produce false-negative results for well-differentiated pulmonary adenocarcinoma [2], or small volumes of metabolically active tumors [3], and false-positive results for inflammatory nodules [4].

For the last two decades, magnetic resonance imaging (MRI) in lung cancer staging has been limitedly used in mediastinum invasion or chest wall invasion of lung cancer after Webb et al. [5] of the Radiologic Diagnostic Oncology Group published results in 1991. Diffusion-weighted magnetic resonance imaging (DWI) has been applied to detect the restricted diffusion of water molecules. The principals of DWI utilize the random motion of water molecules in biologic tissue [6]. Apparent diffusion coefficient (ADC) value is a quantitative parameter of the diffusion of water molecules in biological tissues, and is usually significantly lower in malignant tumors compared with normal tissue or benign lesions [7]. The MR signal intensity of pulmonary cancer nodules was significantly higher than that of benign lesions [8]. A meta-analysis has shown that DWI can be used to differentiate malignant from benign pulmonary lesions [9]. Two articles of meta-analysis reported that DWI was effective for the evaluation of N factor of lung cancer [10, 11]. Peerlings et al. [10] reported high diagnostic capability of DWI for nodal assessment in the non-small cell lung cancer: The sensitivity was 0.87 and the specificity 0.88. DWI can distinguish benign from malignant lesions in the lung [9, 12], in the thorax [13], in the prostate [14], in the breast [15], and in the liver [16].

There were two articles which compared diagnostic capability of DWI with that of FDG-PET/CT for pulmonary nodules and masses [12, 17]: The sensitivity and the accuracy of DWI were significantly higher [12], or the sensitivity of DWI was significantly higher [17] than those of FDG-PET/CT. DWI was reported to be superior to FDG-PET in the detection of primary lesions and the nodal assessment of non-small cell lung cancers [18].

The SUVmax of FDG-PET was reported to be a significant prognostic factor of lung cancer [19]. To our knowledge, there are no articles that have combined FDG-PET/CT and DWI to evaluate the prognostic value of preoperative SUVmax and ADC in lung cancers. The focus of this study is to determine the correlation of SUVmax of FDG-PET/CT and ADC value of DWI with known prognostic factors and to evaluate their prognostic values.

## Materials and methods

### Eligibility

The study protocol for examining DWI and FDG-PET/CT in patients with lung cancer was approved by the ethical committee of Kanazawa Medical University (the approval number: No. 189). Written informed consents for MRI, PET-CT and a pathological examination of resected materials were obtained from each patient after discussing the risks and benefits of the examinations with their surgeons.

### Patients

Two-hundred and twenty-seven patients with primary lung cancer were enrolled in this study. They underwent DWI and PET-CT examination before pulmonary resection with nodal dissection from May 2009 to February 2014. None of the patients had received prior treatment. One-hundred and thirty-four patients were male and 93 were female. Their mean age was 68 years old (range 37–85).

Cell type, cell differentiation, pathological N factor, and the size of the tumor were determined by reviewing the pathology reports. There were 168 adenocarcinomas, 44 squamous cell carcinomas, 5 small cell carcinomas, 3 large cell neuroendocrine carcinoma (LCNEC), 3 large cell carcinomas, and 3 carcinomas of other cell types. TNM classification and the lymph node stations of lung cancer were classified according to the new definition of UICC 7 [20]. There were 113 pathological Stage IA (pStage IA), 49 pStage IB, 20 pStageIIA, 14 pStage IIB, 25 pStage IIIA, 1 pStage IIIB, and 5 pStage IV. There were 77 pathological T1a (pT1a) carcinomas, 42 pT1b carcinomas, 65 pT2a carcinomas, 13 pT2b carcinomas, 25 pT3 carcinomas, and 5 pT4 carcinomas. There were 180 pathological pN0 (pN0) carcinomas, 30 pN1 carcinomas, and 17 pN2 carcinomas.

### PET-CT

FDG-PET scanning was performed with a dedicated PET camera (SIEMENS Biograph Sensation 16, Erlangen Germany) before surgery. All patients fasted for 6 h before scanning. The dose of ^18^F-FDG administered was 3.7 MBq/Kg of body weight. After a 60-min uptake period, an emission scan was acquired for 3 min per bed position and a whole-body scan was performed according to the height of each patient. After image reconstruction, a two-dimensional (2D) round region of interest (ROI) was drawn on a slice after visual detection of the highest count on the fused CT image by a radiologist (N.W.) with 29 years of radioisotope scintigraphy and PET-CT experience who was unaware of the patients’ clinical data. For the lesions with negative or faintly positive PET findings, the ROI was drawn on the fusion image with the corresponding CT. From those ROI, the maximum standardized uptake value (SUVmax) was calculated. The radiologist (N.W.) and one pulmonologist (K.U.) with 28 years of experience evaluated the FDG-PET data. They eventually reached at the same consensus. The optimal cutoff value (OCV) of SUVmax for diagnosing malignancy in PET-CT was determined to be 4.45 using the receiver operating characteristics curve as previously reported [21].

### MR imaging

All MR images were created with a 1.5 T superconducting magnetic scanner (Magnetom Avanto; Siemens, Erlangen, Germany) with two anterior six-channel body phased-array coils and two posterior spinal clusters (six channels each). The conventional MR images consisted of a coronal T1-weighted spin-echo sequence and coronal and axial T2-weighted fast spin-echo sequences. DWIs using a single-shot echo-planar method were performed with slice thickness of 6 mm under SPAIR (spectral attenuated inversion recovery) with respiratory triggered scan with the following parameter: TR/TE/flip angle, 3000-4500/65/90; diffusion gradient encoding in three orthogonal directions; *b* value = 0 and 800 s/mm^2^; field of view, 350 mm; and matrix size, 128 × 128. After image reconstruction, a two-dimensional (2D) round or elliptical region of interest (ROI) was drawn on the lesion which was detected visually on the ADC map with reference to T2-weighted or CT image by a radiologist (M.D.) with 25 years of MRI experience who was unaware of the patients’ clinical data. Areas with necrosis were excluded from the ADC measurement. The procedure was repeated three times, and the minimum ADC value was obtained. The radiologist (M.D.) and one pulmonologist (K.U.) with 28 years of experience evaluated the MRI data. They eventually reached at the same consensus. The OCV of ADC for diagnosing malignancy in DWI was determined to be 1.70 × 10^−3^mm^2^/s using the receiver operating characteristics curve as previously reported [21].

### Statistical analysis

The data are expressed as the mean ± standard deviation. A two-tailed Student t test was used for comparison of ADC values or SUVmax in several prognostic factors. The Kaplan–Meier method was used to calculate the survival rate using death from any cause with a 95% confidence interval (CI), and the log-rank test was used to compare the survival curves. A Cox proportional hazard model was used for the univariate and multivariate survival analyses. The statistical analyses were performed using the computer software program StatView for Windows (Version 5.0; SAS Institute Inc. Cary, NC, USA). A *P* value of < 0.05 was considered statistically significant.

## Results

**Fig. 1:**
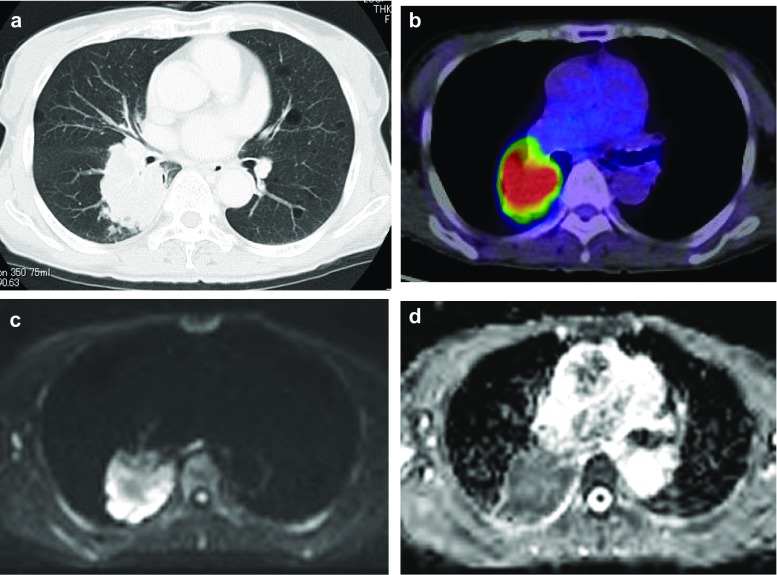
Adenocarcinoma **a** CT, **b** PET-CT, SUVmax 7.79, **c** DWI, and **d** ADCmap, ADC 1.165 × 10^−3^mm^2^/s

**Fig. 2:**
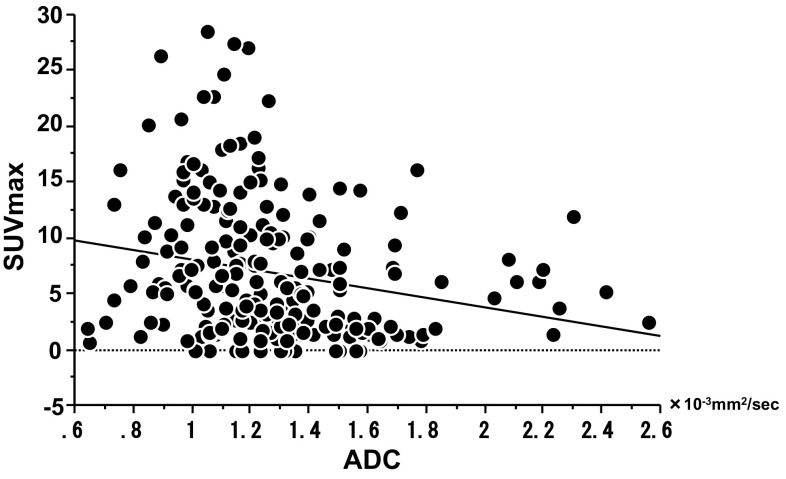
Correlation between SUVmax and ADC of lung cancer. SUVmax = 12.40 − 4.34 × ADC. Correlation coefficient *r* = − 0.227, *P* = 0.0006

Radiological images of a lung cancer (adenocarcinoma) is presented in Fig. 1. There was a significantly weak inverse relationship between SUVmax and ADC (Correlation coefficient *r* = − 0.227, *P* = 0.0006; Fig. 2).

**Fig. 3:**
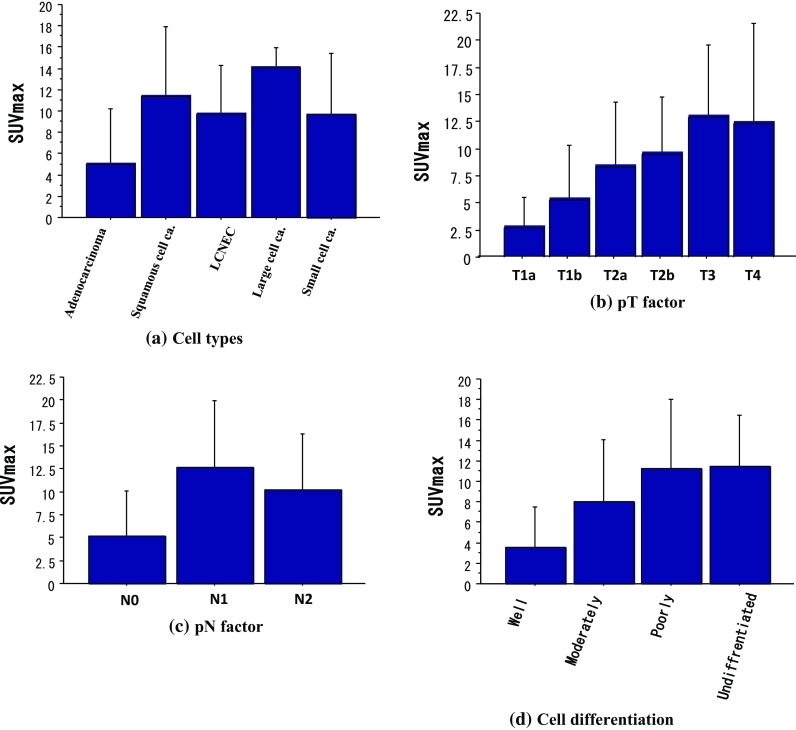
Correlation between SUVmax and several prognostic factors. There were a significant correlation between SUVmax and T factor/cell differentiation

Correlations between the SUVmax and several prognostic factors are presented in Fig. 3. There were a significant correlation between the SUVmax and the pT factor (Fig. 3b). The SUVmax of pN0, pN1, or pN2 lung cancer was 5.13 ± 4.99, 12.67 ± 7.27, or 10.21 ± 6.11, respectively. The SUVmax of pN0 lung cancer was significantly lower than that of pN1 or pN2 lung cancer (*P* < 0.0001; Fig. 3c). There was a significant correlation between the SUVmax and cell differentiation (Fig. 3d). The SUVmax was a factor that is correlated to T factor, N factor, and cell differentiation.

**Fig. 4:**
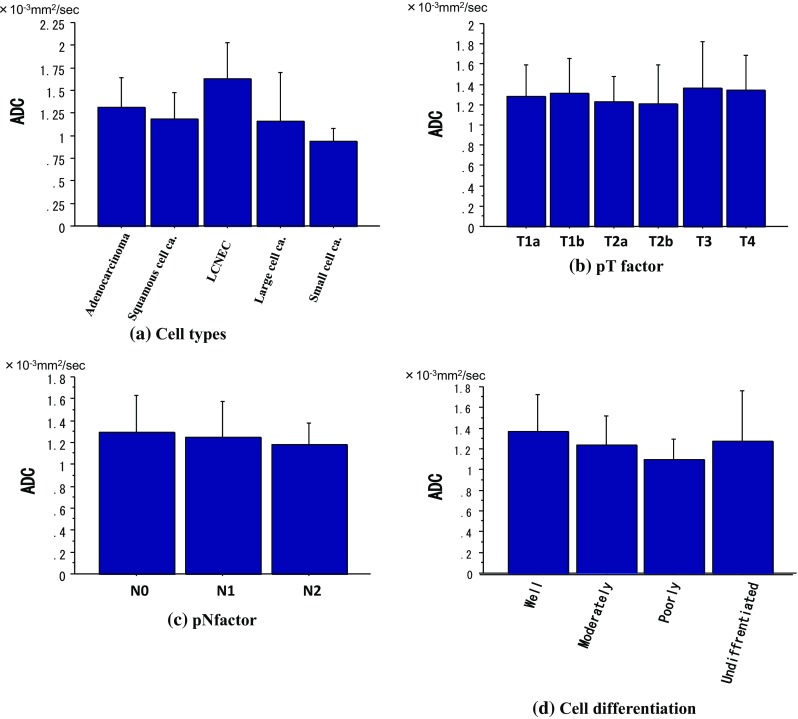
Correlation between ADC and several prognostic factors. There was no correlation between ADC and the several prognostic factors

Correlations between ADC and several prognostic factors are presented in Fig. 4. There was no correlation between ADC and the several prognostic factors (Fig. 4a, b, d). ADC of pN0, pN1, or pN2 lung cancer was 1.29 ± 0.34, 1.24 ± 0.33, 1.17 ± 0.20 × 10^−3^mm^2^/s, respectively (Fig. 4c). ADC of lung cancer is a factor that is not correlated to T factor, or N factor.

**Fig. 5:**
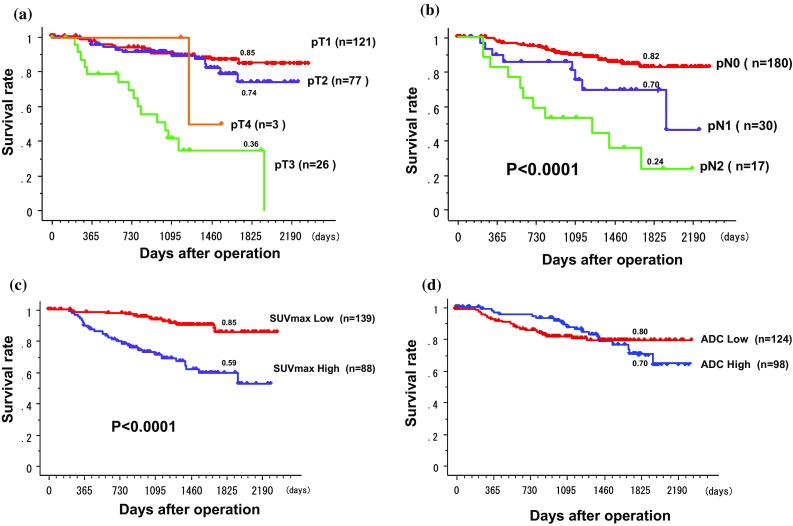
**a** Survival rates by pT factor. There were significant differences among them (*P* < 0.0001). **b** Survival rates by pN factor. There were significant differences among them (*P* < 0.0001). **c** Survival rates by SUVmax. SUVmax was divided into two groups by the mean value of 6.50. The 5-year survival rate (0.85) of patients in the SUVmax low group where the SUVmax was under 6.50 was significantly higher than that (0.59) of patients in the SUVmax high group where the SUVmax was 6.50 or higher (*P* < 0.0001). **d** Survival rates by ADC. ADC was divided into two groups by the mean value of 1.27 × 10^−3^mm^2^/s. The 5-year survival rate (0.80) of patients in the ADC low group where the ADC was under 1.27 × 10^−3^mm^2^/s was not higher than that (0.70) of patients in the ADC high group where the ADC was 1.27 × 10^−3^mm^2^/s or higher (*P* = 0.768)

Survival curves by several factors are presented in Fig. 5. Five-year survival rate (0.89) of female patients was significantly higher than that (0.67) of male patients (*P* = 0.0037). Five-year survival rate (0.85) of patients under 70 years old was significantly higher than that (0.65) of patients 70 years old or older (*P* = 0.0160). For survival rates by pT factor, there were significant differences among them (*P* < 0.0001; Fig. 5a). For survival rates by pN factor, 5-year survival rates of pN0, pN1, or pN2 lung cancer were 0.82, 0.70, or 0.24, respectively. There were significant differences among them (*P* < 0.0001; Fig. 5b). For survival rates by cell differentiation, there were significant differences among them (*P* = 0.0054). For survival rates by cell type, 5-year survival rate (0.80) of patients with adenocarcinomas was significantly higher than that (0.62) of patients with other cell types (*P* = 0.0003). The SUVmax was divided into two groups by the mean value of 6.50. The 5-year survival rate (0.85) of patients in the SUVmax low group with an SUVmax under 6.50 was significantly higher than that (0.59) of patients in the SUVmax high group with an SUVmax of 6.50 or more (*P* < 0.0001). (Figure 5c). ADC was divided into two groups by the mean value of 1.27 × 10^−3^mm^2^/s. The 5-year survival rate (0.80) of patients in the ADC low group with an ADC under 1.27 × 10^−3^mm^2^/s was not higher than that (0.70) of patients in the ADC high group with an ADC of 1.27 × 10^−3^mm^2^/s or more (*P* = 0.768; Fig. 5d).

Univariate analysis using a Cox proportional hazard model revealed that SUVmax, pN factor, age, cell differentiation, cell type, sex, and pT factor were significant (Table 1). But ADC was not a significant factor. The multivariate analysis of factors influencing survival by a Cox proportional hazard model revealed that independent significant prognostic factors were SUVmax (*P* = 0.0202) and pN factors (*P* = 0.0353), and ADC was not (*P* = 0.0581; Table 2).

**Table 1:** Univariate analysis

Factor		Hazard ratio	95% CI		*P* value
SUVmax	Low/high	4.485	2.325	8.652	< 0.0001
pN factor	pN0/pN1-2	0.257	0.14	0.473	< 0.0001
ADC	Low/high	1.108	0.599	2.05	0.7426
Age	Under 70 years old/70 years old or more	2.086	1.112	3.914	0.0219
Cell differentiation	Well/moderately ~ undifferentiated	0.329	0.165	0.656	0.0016
Cell type	Adenocarcinoma/other cell types	0.352	0.191	0.646	0.0008
Sex	Female/male	0.354	0.169	0.743	0.006
pT factor	pT1/pT2-4	0.381	0.2	0.724	0.0033

**Table 2:** Multivariate analysis

Factor		Hazard ratio	95% CI		P value
SUVmax	Low/high	2.728	1.17	6.363	0.0202
pN factor	pN0/pN1-2	0.469	0.231	0.949	0.0353
ADC	Low/high	1.939	0.977	0.3846	0.0581
Age	Under 70 years old/70 years old or more	1.714	0.888	3.308	0.108
Cell differentiation	Well/moderately ~ undifferentiated	0.611	0.266	1.399	0.2436
Cell type	Adenocarcinoma/other cell types	0.698	0.335	1.454	0.3369
Sex	Female/male	0.687	0.306	1.541	0.3625
pT factor	pT1/pT2-4	0.997	0.449	2.212	0.9934

## Discussion

Our main finding was that SUVmax of lung cancer has a stronger relationship with known prognostic factors and may be more useful for predicting the prognosis of lung cancer than ADC values of lung cancer. Although ADC of DWI is useful to distinguish benign from malignant lesions in the lung, ADC itself is not a significant prognostic factor in lung cancer and is not related to known prognostic factors. The difference found in prognostic significance can be explained by the fact that FDG-PET provides quantitative information regarding cellular glucose metabolism which is associated to tumor aggressiveness, while DWI provides quantitative information regarding tissue cellularity and the diffusion of water molecules which are not necessarily associated to tumor aggressiveness. In our study, there was a significantly weak inverse relationship between ADC and SUVmax. It may mean that the higher SUVmax of lung cancer is, the lower the corresponding ADC is. Although SUVmax and ADC represent different aspects of the biologic features of the tumor, SUVmax showing metabolic activity may be correlated to ADC showing tumor cellularity and diffusion of water.

In breast cancer, Karan et al. [22] and Kitajima et al. [23] reported that SUVmax was significantly associated with known prognostic factors such as tumor size, histological grade, lymph node status, estrogen receptor status, human epidermal growth factor receptor 2 status, whereas ADC values were not, which concluded that SUVmax may be valuable for predicting the prognosis of breast cancer. This result is similar to our data. On the other hand, Nakajo et al. [24] and Choi et al. [25] mentioned that SUVmax and ADC correlated with several pathological prognostic factors and both indexes may have the same potential for predicting the prognosis of breast cancer. DWI-MRI and FDG-PET/CT have their own advantages [26]. Gallivanone et al. [27] reported that FDG-PET predicts patient prognosis and DWI predicts response to neoadjuvant chemotherapy, and both examinations provide useful complementary information for biological characterization and neoadjuvant chemotherapy response prediction in breast cancer.

We have to keep in mind that there are two important limitations in this study. First, it had a retrospective study and was conducted at a single institution, which would have unavoidably introduced selection bias. Second, our ADC measurements were repeated three times and the minimum ADC value was obtained. This ADC may not be fully representative for the whole tumor. There is no consensus in the literature concerning the optimal DWI techniques and image analysis procedure, including ROI size and placement.

Concerning survival of patients with locally advanced non-small cell lung cancer, staging with FDG-PET/CT was reported to be superior to conventional staging methods [28]. DWI was reported to have better potential than FDG-PET/CT for prediction of tumor response to therapy in non-small cell lung cancer patients before chemoradiotherapy [29]. Further studies are necessary to evaluate the performance of FDG-PET/CT and DWI for treatment and survival of lung cancer patients.

## Conclusion

SUVmax is a significant prognostic factor that is correlated to known prognostic factors. But ADC of DWI is not correlated to these factors and not a significant prognostic factor. SUV max may be more useful for predicting the prognosis of lung cancer than ADC values.

## Abbreviations

FDG-PET: Positron emission tomography with 18-fluoro-2-deoxy-glucose
DWI: Diffusion-weighted magnetic resonance imaging
SUVmax: Maximum standardized uptake value
MRI: Magnetic resonance imaging
ADC: Apparent diffusion coefficient
LCNEC: Large cell neuroendocrine carcinoma
ROI: Region of interest
OCV: Optimal cutoff value
SPAIR: Spectral attenuated inversion recovery
